# Beyond ovarian morphology: What the renaming of polycystic ovary syndrome means for dermatology

**DOI:** 10.1016/j.jdin.2026.06.015

**Published:** 2026-06-30

**Authors:** Umayr R. Shaikh, Anya Chan, Bouchra Benghomari, Rida Saeed, H. William Higgins, Jane M. Grant-Kels

**Affiliations:** a University of Pennsylvania Department of Dermatology, Philadelphia, Pennsylvania; b Georgetown University School of Medicine, Washington, DC; c Tufts University, Medford, Massachusetts; d University of Connecticut Department of Dermatology, Farmington, Connecticut; e University of Florida Health Department of Dermatology, Gainesville, Florida

**Keywords:** metabolic syndrome, nomenclature, polycystic ovary syndrome, polyendocrine metabolic ovarian syndrome, racial disparities, women’s health

*To the Editor:* An international consensus has renamed polycystic ovary syndrome, also known as PCOS, to polyendocrine metabolic ovarian syndrome (PMOS), with patients and health professionals agreeing that PMOS better recognizes the endocrine, hormonal, and metabolic nature of the disease.^1^ This renaming reflects a broader conceptual shift away from an ovarian-centric understanding of the condition toward recognition of its systemic endocrine and metabolic manifestations and offers an opportunity to reconsider the disease through the lens of what drives long-term morbidity. Because dermatologic findings often precede diagnosis, dermatologists are uniquely positioned to operationalize this shift through earlier recognition, metabolic risk assessment, and population-aware screening practices. Our purpose is not to evaluate whether PMOS represents the ideal nomenclature but rather to examine what this renaming means for dermatologic practice.

The populations most affected by PMOS are often those least likely to receive a timely diagnosis. In a recent analysis of >20,000 individuals at risk, Black patients had 69% higher odds of a missed diagnosis than White patients, while individuals receiving Medicaid or charity coverage faced nearly twice the odds of underdiagnosis, with underdiagnosis tracking closely with neighborhood-level social vulnerability.^2^ Once diagnosed, important differences in disease expression and burden have also been documented across populations. Black women experience higher rates of metabolic syndrome and insulin resistance, while racial and ethnic differences in hyperandrogenic and metabolic burden have also been documented across populations with PMOS.^3^ These observations highlight the importance of population-aware diagnostic approaches and the recognition that disease presentation and downstream morbidity may vary across populations. By foregrounding endocrine and metabolic features in the name rather than ovarian pathology, PMOS better reflects the multisystem nature of the disorder, including insulin resistance, obesity, metabolic syndrome, type 2 diabetes, and cardiovascular risk.^1^ Importantly, this broader framing does not diminish the significance of reproductive manifestations, such as ovulatory dysfunction and infertility, but instead situates them within a systemic endocrine-metabolic disease process.^1^ The broader conceptual shift accompanying the renaming may also reinforce the importance of population-aware diagnostic approaches, an area dermatology has increasingly begun to address. The 2023 International Evidence-Based Guideline recalibrated modified Ferriman-Gallwey thresholds according to ethnicity, acknowledging that diagnostic tools validated primarily in White European populations may not perform equally across all groups.^4^

Dermatology is well positioned to operationalize this evolving framework. Cutaneous manifestations, including hirsutism, acne, acanthosis nigricans, and androgenetic alopecia, are often among the first presenting complaints in PMOS, and dermatology clinics are frequently among the earliest specialty contacts before metabolic sequelae accumulate.^5^ If PMOS is increasingly understood as a systemic endocrine-metabolic disorder, then dermatologists can contribute meaningfully by advocating for population-aware diagnostic thresholds, incorporating metabolic screening considerations into the evaluation of androgenic skin findings, and participating in multidisciplinary conversations surrounding equitable PMOS care. Earlier recognition of PMOS in dermatology settings may facilitate timely referral, metabolic risk assessment, and intervention before the development of long-term complications. The renaming of polycystic ovary syndrome to PMOS may not itself resolve longstanding disparities, but it provides an opportunity to rethink how clinicians recognize and evaluate a disease whose earliest manifestations frequently present in dermatology clinics.

## Conflicts of interest

None disclosed.
